# Volumes of hippocampal subfields suggest a continuum between schizophrenia, major depressive disorder and bipolar disorder

**DOI:** 10.3389/fpsyt.2023.1191170

**Published:** 2023-07-20

**Authors:** Peiyu Cao, Congxin Chen, Qi Si, Yuting Li, Fangfang Ren, Chongyang Han, Jingjing Zhao, Xiying Wang, Guoxin Xu, Yuxiu Sui

**Affiliations:** ^1^Department of Psychiatry, The Affiliated Brain Hospital of Nanjing Medical University, Nanjing Brain Hospital, Nanjing, China; ^2^Women’s Hospital of Nanjing Medical University, Nanjing Maternity and Child Health Care Hospital, Nanjing, China; ^3^Huai’an No. 3 People’s Hospital, Huai’an, China

**Keywords:** hippocampal subfields, psychosis continuum, schizophrenia, bipolar disorder, major depressive disorder, MRI

## Abstract

**Objective:**

There is considerable debate as to whether the continuum of major psychiatric disorders exists and to what extent the boundaries extend. Converging evidence suggests that alterations in hippocampal volume are a common sign in psychiatric disorders; however, there is still no consensus on the nature and extent of hippocampal atrophy in schizophrenia (SZ), major depressive disorder (MDD) and bipolar disorder (BD). The aim of this study was to verify the continuum of SZ – BD – MDD at the level of hippocampal subfield volume and to compare the volume differences in hippocampal subfields in the continuum.

**Methods:**

A total of 412 participants (204 SZ, 98 MDD, and 110 BD) underwent 3 T MRI scans, structured clinical interviews, and clinical scales. We segmented the hippocampal subfields with FreeSurfer 7.1.1 and compared subfields volumes across the three diagnostic groups by controlling for age, gender, education, and intracranial volumes.

**Results:**

The results showed a gradual increase in hippocampal subfield volumes from SZ to MDD to BD. Significant volume differences in the total hippocampus and 13 of 26 hippocampal subfields, including CA1, CA3, CA4, GC-ML-DG, molecular layer and the whole hippocampus, bilaterally, and parasubiculum in the right hemisphere, were observed among diagnostic groups. Medication treatment had the most effect on subfields of MDD compared to SZ and BD. Subfield volumes were negatively correlated with illness duration of MDD. Positive correlations were found between subfield volumes and drug dose in SZ and MDD. There was no significant difference in laterality between diagnostic groups.

**Conclusion:**

The pattern of hippocampal volume reduction in SZ, MDD and BD suggests that there may be a continuum of the three disorders at the hippocampal level. The hippocampus represents a phenotype that is distinct from traditional diagnostic strategies. Combined with illness duration and drug intervention, it may better reflect shared pathophysiology and mechanisms across psychiatric disorders.

## Introduction

The lifetime prevalence of psychosis spectrum is approximately 3%, where the lifetime risk of schizophrenia (SZ) is 1%, while the lifetime prevalence of recurrent affective disorders, such as major depressive disorder (MDD) and bipolar disorder (BD) is 16 and 1%, respectively (1–4). These severe disorders affect millions of people worldwide and are at the forefront of global disease burden due to stigma, activity limitation, decreased life expectancy, and increased medical expenditures. Previous studies have found overlaps in many aspects in SZ, MDD, and BD. For example, white matter integrity deficits and gray matter reductions, both in magnitude and spatial, were present in SZ and BD (5). Abnormalities in white matter connectivity and white matter hyperintensities were present in MDD and BD (6). From the perspective of symptomatology, core aspects of SZ, such as hallucination and delusion, speech and thought disorganization, apathy, and cognitive dysfunction, are experienced by approximately 50% of patients with BD during episodes (7). On the other hand, negative symptoms of SZ are often difficult to distinguish from depressive episodes of BD and MDD, especially because 40% of patients with SZ have comorbid depressive disorders (8). Indeed, most patients do not fully meet the diagnostic parameters and exhibit a mixture of clinical manifestations of the three disorders. In clinical practice, differential diagnosis has been proven difficult. Currently, it remains unclear whether the three disorders are categorical or continuous.

The notion of continuous psychiatric disorders involves the development of the concept of “unitary psychosis,” which is used to explain the nonarbitrary boundary between schizophrenia and other psychiatric disorders (9, 10). Since then, the concept has been refined and has attracted increasing attention. Crow (11) concluded that affective psychoses and schizophrenia are related on a continuum and deduced a case for a psychosis continuum that increases in severity from unipolar to bipolar affective disorder to schizoaffective disorders and schizophrenia with increasing degree of deficits. Considering SZ, MDD and BD as a meaningful continuum comes from several sources beyond symptomatology, including their family aggregation and genetic overlaps (12–14). Some genes may have certain influence beyond diagnostic boundaries and may also show pleiotropic and disorder-specific effects across the three disorders (15). In addition, similarities in the patterns of cytokine alterations in the three disorders during acute and chronic phases of disease raise the possibility of a common pathology in immune dysfunction (16). Finally, they were found to have certain mechanisms for antipsychotics and other treatments (17). There have been several attempts to establish transdiagnostic and dimensional approaches for treatment decisions, such as the Research Domain Criteria (RDoC) project, the Hierarchical Taxonomy of Psychopathology (HiTOP), and the Clinical High At Risk Mental State (CHARMS) approach (18). Beyond that, previous studies have been devoted to demonstrating different psychosis continua. In their study, Benazzi et al. (19, 20) reported no-bimodality distribution of intradepressive hypomanic symptoms between BP-II and MDD using systematically assessed symptoms, supporting the spectrum view of mood disorders. Another study focusing on the continuum hypothesis of SZ and BD found a shared psychotic core in a distributed network involving parts of medial parietal and temporo-occipital areas, as well as parts of the cerebellum and middle frontal gyrus, at the neuroimage level (21). Similar studies involving the hippocampus have also been considered (22).

The hippocampus is located in the deep medial temporal lobe and is one of the most important components of the limbic system (23). It consists of the dentate gyrus (DG) and the cornu Ammonis (CA) area, connected with the subiculum, which extends down to the entorhinal cortex, and together forms the hippocampus (24, 25). Hippocampus is involved in cognition, learning, memory acquisition and consolidation, and declarative memory extraction, in addition to having key roles in emotion regulation, motivated behavior, and neuroendocrine stress responses, all of which are impaired to varying degrees in SZ, MDD, and BD. In studies using deep learning for subtype classification of major psychiatric disorders, risk genes were found to be significantly expressed in hippocampal tissues of atypical psychiatric disorders, suggesting that the hippocampus has shared genetic risk genes in SZ, MDD and BD that could serve as potential genetic biomarkers for psychiatric disorders (26–29). Recently, an increasing number of studies have found that the hippocampus is associated with pathology and dysfunction in psychosis spectrum disorders. Reduced hippocampal volume is associated with increased blood perfusion, reduced activation in memory tasks, symptom severity, social function, and antipsychotics effects (30–34). Studies using fMRI have also highlighted the hippocampal alterations in traditional diagnostic groups (35–37). Regarding transdiagnostic fMRI findings, duration of brain networks involved in emotion processing, which includes the hippocampus, showed an increase in a transdiagnostic sample of patients with MDD and BD, emphasizing the importance of the hippocampus in these disorders from a multimodal perspective (38). Considering the function of hippocampus, these alterations may contribute to the pathogenesis and cognitive impairment in psychosis continua. So far, the hippocampus has been identified as one of the most valuable structures for human brain research.

From a neural perspective, previous studies have detected overlapping alterations in the hippocampus in SZ, MDD, and BD, supporting the hypothesis of continuum at the hippocampal level. Several studies have demonstrated that both SZ and MDD exhibit atrophy in CA1 and CA4/DG compared to healthy controls (39–41). In SZ and BD, overlapping alterations were found in CA2/3 and CA4/DG, whereas in MDD and BD, overlapping alterations were found in CA4/DG and subiculum (39–42). When directly compared with each other, the alterations in hippocampal subfields were different. There were no significant differences in hippocampal volume between MDD and BD (41, 43). Smaller left CA2/3, right presubiculum and bilateral subiculum were found in SZ than in BD (44). Smaller CA and DG were found in SZ than in MDD (45). Therefore, anatomical evidence suggests that hippocampal deficits differ in severity in the three disorders: atrophy is more severe in some subfields in SZ, while it is less severe in MDD and BD. In fact, hippocampal atrophy in BD seems to be an intermediate level between SZ and MDD.

Despite notable results, the conclusions are limited. Most studies have only compared patients with healthy controls and indirectly yielded the hippocampal relationships between patient groups by analyzing their relationships with healthy controls. Besides, we found few research concentrating on the continuum hypothesis of SZ, MDD, and BD, while there were quite a few direct three-group comparisons of hippocampus formation (46). Alterations of hippocampal and hippocampal subfield volumes between them remain unclear. In addition, there is notable heterogenicity in previous studies, such as diagnostic criteria, hippocampus segmentation tools, imaging instruments, and potential confounding or modifying factors (e.g., drugs, illness duration, and disease state).

Considering these limitations, we aimed to test the hypothesis of the continuum of SZ, MDD and BD at the hippocampal level, referring to a slight to a severe loss of hippocampal subfield volumes. We hypothesized that there would be a continuum of psychosis from MDD to BD to SZ, with increasing severity and decreasing hippocampal subfield volumes. We also compared the effect of the presence or absence of medication treatment on hippocampal subfield volumes. We hypothesized that patients without antipsychotic medication would have smaller subfield volumes than patients with antipsychotic medication. Illness duration, as well as drug dose, were hypothesized to be associated with volume reductions in the hippocampus. We hypothesized that the illness duration as well as drug dose would be associated with volume reductions in hippocampal subfields.

## Methods

### Participants

261 SZ, 126 MDD, and 155 BD individuals according to the criteria listed in the fifth edition of the Diagnostic and Statistical Manual of Mental Disorders (DSM-5) were recruited from the Department of Psychiatry, the Affiliated Brain Hospital of Nanjing Medical University (47). Exclusive criteria included the history of traumatic head injury, neurological disease or tumor, history of substance dependence or abuse, comorbidity with any other psychiatric disorders, left-handedness, and the presence of any MRI-identified brain abnormality or microvascular lesion on T1 or T2-weighted images.

Initial diagnoses of potential participants were made by their treating psychiatrists, who provided all available clinical information on participants. Two senior psychiatrists then independently performed confirmatory diagnoses using the DSM-5. When diagnostic disagreement occurred between the treating and two senior psychiatrists, the participant was excluded from the sample. From the initial sample, 40 patients were excluded due to diagnostic disagreement, 16 due to the lack of T2-weighted image, 45 due to poor image quality, 6 due to a history of substance abuse, 17 due to comorbidity with other psychiatric disorders, and 6 due to comorbidity with organic brain diseases. The remaining participants were 204 SZ, 98 MDD and 110 BD.

To evaluate current clinical symptoms in each group, Brief Psychiatric Rating Scale (BPRS) was used in SZ group and patients with psychotic symptoms in MDD and BD groups, Hamilton Rating Scale for Anxiety (HAMA) and version of 24-item Hamilton Rating Scale for Depression (HAMD) were used in MDD group and patients in depressive episodes in BD group, and Young Mania Rating Scale (YMRS) was used in BD group (48–50). The clinical questionnaires were administered by patients’ treating psychiatrists and confirmed by the two senior psychiatrists, same as the diagnostic strategy. The study was approved by the Ethics Committee of the Affiliated Brain Hospital of Nanjing Medical University. All participants accepted and signed a written informed consent after a complete explanation of the study procedures.

### MRI data acquisition and processing

**Figure 1:**
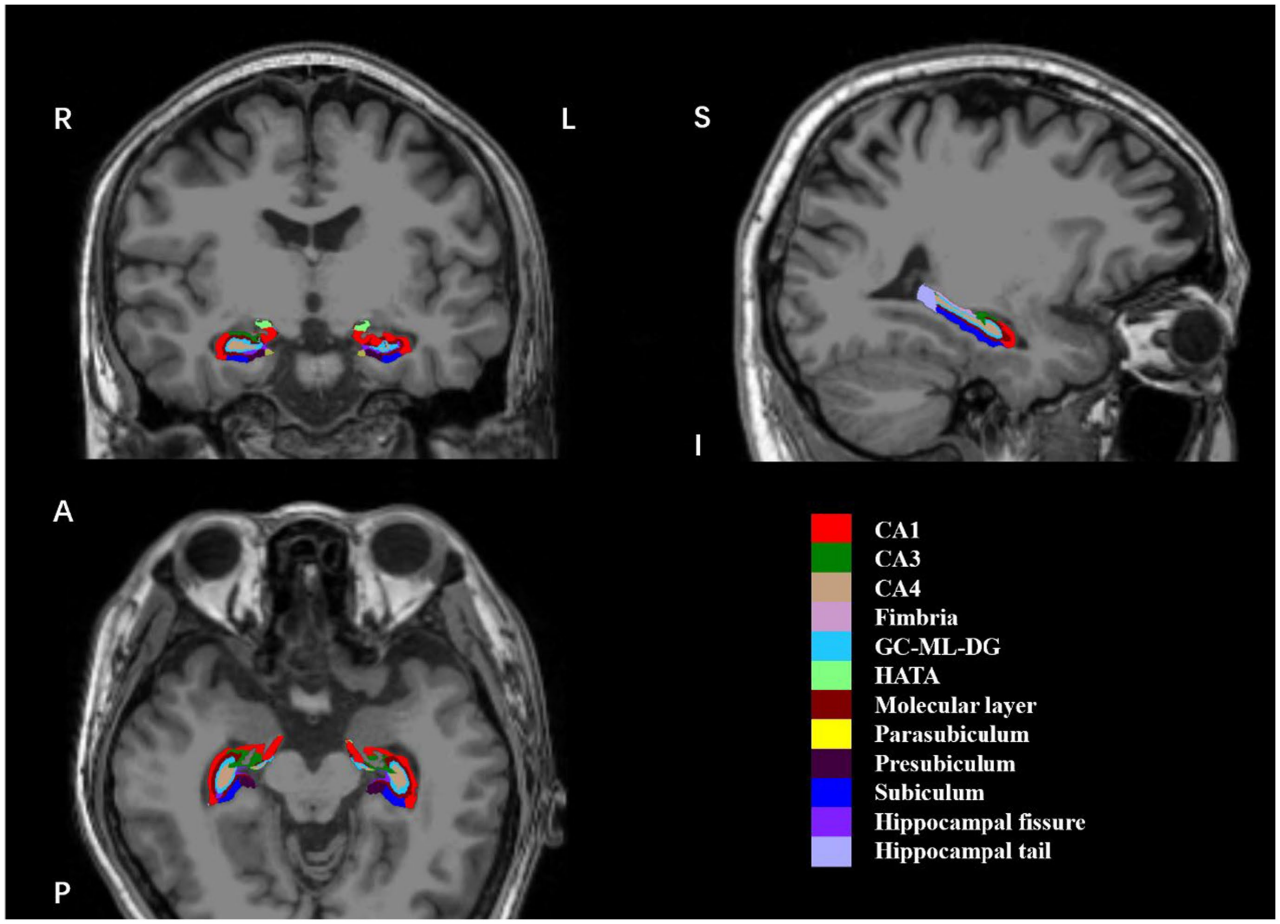
Coronal, sagittal and axial views of 12 hippocampal subfields. The hippocampal subfield volumes were overlaid on whole-brain T1-weighted images in a specific participant in this study. These images were displayed using FreeView. R, right; L, left; (S), superior; I, inferior; (A), anterior; (P), posterior; CA, cornu ammonis; GC-ML-DG, granule cells in the molecular layer of the dentate gyrus; HATA, hippocampal-amygdaloid-transition-area.

All participants were examined on a 3.0 T GE scanner at Nanjing Brain Hospital using a 3D T1weighted GR sequence (TE = 3.192 ms; TR = 8.24 ms; flip angle = 12°; matrix = 256 × 256; FOV = 100 mm; slice thickness = 1.0 mm; 1.0 × 1.0 × 1.0 mm voxels; 176 slices). 3D T2 weighted image were obtained using SE sequence (TE = 106.56 ms; echo train length 32, 142° flip angle, matrix = 512 × 512; 0.47 × 0.47 mm in plane resolution, 8 mm slice thickness). To extract hippocampal subfield volumes, we used an automated segmentation algorithm for sMRI, which was released as part of the latest FreeSurfer v7.1.1 (51). It provides anatomically cortical reconstruction and volumetric segmentation of 12 subfields of the hippocampus, including CA1, CA3 (with CA2 included), CA4, fimbria, granule cells in the molecular layer of the DG (GC-ML-DG), hippocampal-amygdaloid transition area (HATA), molecular layer (ML), subiculum, presubiculum, parasubiculum, hippocampal fissure, and hippocampal tail (Figure 1). Total hippocampal volumes and estimated total intracranial volumes (eTIV) were also automatically extracted according to the algorithm, and eTIV was used to represent intracranial volumes (ICV) for follow-up statistics. The segmentation procedure was fully automated by recon-all pipeline and step segmentHA_T2, without manual editing (51). After visual inspection, no undesirable segmentation was observed in any subject.

### Statistical analyses

SPSS version 26.0 was used for all statistical analyses. We used the Shapiro-Wilk test with statistical significance set at *p* < 0.05 to analyze the normality of the values of demographic and clinical variables, including age, illness duration, education, onset age, drug dose equivalent, BPRS, HAMA, HAMD, YMRS, ICV, and subfield volumes. The distribution of age, education, onset age, BPRS, HAMA, HAMD, YMRS, ICV, and subfield volumes showed normal distributions. The other parameters showed non-normal distributions. To examine the differences in age, education, onset age, and ICV between the three groups, the analysis of variance (ANOVA) was used. To examine the differences in gender between the three groups, we used the Chi-square test. To examine the differences in illness duration, we used the Kruskal-Wallis test. A Bonferroni procedure (adjusted *p* = *p* × 3 for groups) was performed for *post-hoc* comparison between two groups. An adjusted *p*-value was reported throughout when a test was significant. Adjusted *p* < 0.05 was deemed significant.

The hippocampal subfield volumes differences across groups were compared employing multivariate analysis of covariance (MANCOVA), with subfields as dependent variables, diagnosis as a fixed factor, and age, gender, education, and ICV as covariates. A Bonferroni procedure (adjusted *p* = *p* × 26 for hippocampal subfield volumes) was performed for *post-hoc* comparison between two groups. Total hippocampal volume was tested using analysis of covariance (ANCOVA) with the same parameters and *p*-values as for subfield analysis. Using the same statistical method as for the main analysis, we performed subgroup analyses comparing hippocampal subfield volumes between patients with and without medication treatment in all groups. The significance threshold was set at adjusted *p* < 0.05.

As the BD group contained both BD-I (*n* = 92) and BD-II (*n* = 18) patients, we compared the differences in hippocampal subfield volumes between SZ, MDD, and the two subgroups using the same statistical method applied in the main analysis to evaluate the effect of BD type. A Bonferroni procedure (adjusted *p* = *p* × 26 for hippocampal subfields) was performed for *post-hoc* comparison between two groups. Since the lithium has been reported to have neuroprotective effects in the hippocampus, lithium usage should be considered as a confounding factor (52). We compared the differences in hippocampal subfield volumes between lithium-treated (*n* = 63) and non-treated (*n* = 47) patients with BD using the same MANCOVA analysis with significance set at adjusted *p* < 0.05 (adjusted *p* = *p* × 26 for hippocampal subfields). Regarding the influence of illness duration and medication on hippocampal subfields, we examined the correlation of hippocampal subfield volumes with illness duration and drug dose equivalent in each group by Kendall’s tau-b correlation, because the data were not normally distributed and there were multiple identical values for illness duration and drug dose. Drug dose correlations excluded drug-naïve patients, leaving 160 SZ, 69 MDD, and 98 BD. To quantify the hemispherical asymmetry of the subfields, the following formula was used: (right–left)/ (right+left). The index ranged from −1 to 1, with positive values indicating a larger volume of right subfield. Sets of paired *t*-tests were used for subfield volume comparisons between hemispheres, with a significance threshold set at *p* < 0.05. A MANCOVA was performed with the same parameters as the main analysis to compare the cross-sectional differences in the laterality of hippocampal subfield volumes between the three groups, with significance set at adjusted *p* < 0.05 (adjusted *p* = *p* × 13 for paired hemispheres). A complemental analysis of the correlation between 7 factors of HAMD scores and hippocampal subfield volumes in MDD group was set to explore the influence of clinical symptoms on hippocampal volumes using Pearson’s partial correlation analysis, controlling for age, gender, education, and ICV. Statistical significance was set at *p* < 0.05.

## Results

### Demographic and clinical data of participants

Demographic and clinical variables are presented in Table 1. A total of 204 patients with SZ (102 males and 102 females), 98 patients with MDD (24 males and 74 females), and 110 patients with BD (32 males and 78 females) were included in this study. Twenty-four patients with MDD (24.49% of MDD) and 29 patients with BD (26.36% of BD) presented with psychotic features. In BD group, 32 were in a depressive episode, 69 in a hypomanic or manic episode, and 9 in a mixed episode. Ninety-two BD were diagnosed as BD-I and 18 BD-II. Among all participants, 85 patients (20.63%) had never received any psychotropic medication, while the remaining 327 patients (79.36%) had received it. At the scanning time point, 368 patients were receiving antipsychotics (89.32%), 131 patients (31.80%) were receiving antidepressants, 78 patients (18.93%) were receiving lithium, and 58 patients (14.08%) were receiving antiepileptics. ANOVA for the three groups showed significant differences in age (*F* = 5.29, adjusted *p* = 5.42 × 10^−3^), education (*F* = 4.07, adjusted *p* = 1.78 × 10^−2^), onset age (*F* = 10.79, adjusted *p* = 2.70 × 10^−5^), and ICV (*F* = 5.24, adjusted *p* = 5.67 × 10^−3^). *Post-hoc* analysis showed that mean age and onset were significantly higher in SZ than in BD, and ICV was significantly larger in SZ than in BD and MDD. Kruskal-Wallis test showed a significant difference in illness duration (*H* = 49.36, adjusted *p* = 1.91 × 10^−11^), which was significantly longer in SZ and BD than in MDD.

**Table 1:** Demographic and clinical characteristics of each diagnostic group.

Clinical values	SZ (*n* = 204)	MDD (*n* = 98)	BD (*n* = 110)	*F, H* or *χ*^2^	adjusted *p* values	*post hoc*
Age, mean ± SD, years	34.65 ± 11.48	31.16 ± 14.07	30.40 ± 11.96	5.29	**5.42 × 10** ^**−3**^	SZ > BD
Gender (male/female)	102/102	24/74	32/78	23.66^a^	**7.00 × 10** ^**−6**^	–
Education, mean ± SD, years	12.61 ± 3.31	12.34 ± 3.41	13.52 ± 2.91	4.07	**1.78 × 10** ^**−2**^	BD > MDD
DOI, median (IQR), years	5.00 (10.00)	1.46 (3.63)	5.00 (11.00)	49.36^b^	**1.91 × 10** ^**−11**^	SZ, BD > MDD
Onset age, mean ± SD, years	26.26 ± 9.82	28.06 ± 13.50	21.77 ± 7.52	10.79	**2.70 × 10** ^**−5**^	SZ > BD
ICV, mean ± SD, mm^3^	1480728.40 ± 165617.11	1418163.28 ± 167769.28	1428297.15 ± 215568.24	5.24	**5.67 × 10** ^**−3**^	SZ > MDD, BD
BD-I/BD-II, *n*	–	–	92/18	–	–	–
Psychotic patients, *n*	204	24	29	–	–	–
Current mood status						
Manic/hypomanic, *n*	–	–	69	–	–	–
Depressive, *n*	–	98	32	–	–	–
Mixed, *n*	–	–	9	–	–	–
Drug-treated patients, *n*	160	69	98	–	–	–
Medication						
Antipsychotic, *n*	204	57	107	–	–	–
Antidepressant, *n*	14	95	22	–	–	–
Lithium, *n*	2	12	64	–	–	–
Antiepileptic, *n*	6	1	51	–	–	–
Antipsychotic, median (IQR), mg/d^c^	450.45 (270.60)	150.15 (37.50)	300.30 (250.01)	–	–	–
Antidepressant, median (IQR), mg/d^d^	–	40.00 (11.11)	–	–	–	–
Lithium, median (IQR), mg/d	-	-	600.00 (600.00)	–	–	–
BPRS, mean ± SD	49.12 ± 11.13	43.83 ± 2.78	45.03 ± 5.08	–	–	–
HAMA, mean ± SD	–	19.88 ± 7.27	19.28 ± 4.73	–	–	–
HAMD - 24, mean ± SD	–	32.00 ± 8.51	29.59 ± 6.83	–	–	–
YMRS, mean ± SD	–	–	31.49 ± 9.12	–	–	–

Bonferroni correction was applied: adjusted *p* = *p* × 3.

SZ, patients with schizophrenia; MDD, patients with major depression disorder; BD, patients with bipolar disorder; BPRS, Brief Psychiatric Rating Scale; HAMA, Hamilton Rating Scale for Anxiety; HAMD, Hamilton Rating Scale for Depression; YMRS, Young Mania Rating Scale; R, right; L, left; DOI, duration of illness; ICV, intracranial volume; SD, standard deviation; IQR, interquartile range. Means ± SDs and media (IQR) are shown.

a χ^2^ test. *p* values < 0.05 are shown in boldface.

b Kruskal-Wallis test. *p* values < 0.05 are shown in boldface.

c 100 mg/d chlorpromazine (CPZ) equivalents.

d 40 mg/d fluoxetine (FLX) equivalents.

### Volume differences of the hippocampus and hippocampal subfields across groups

**Figure 2:**
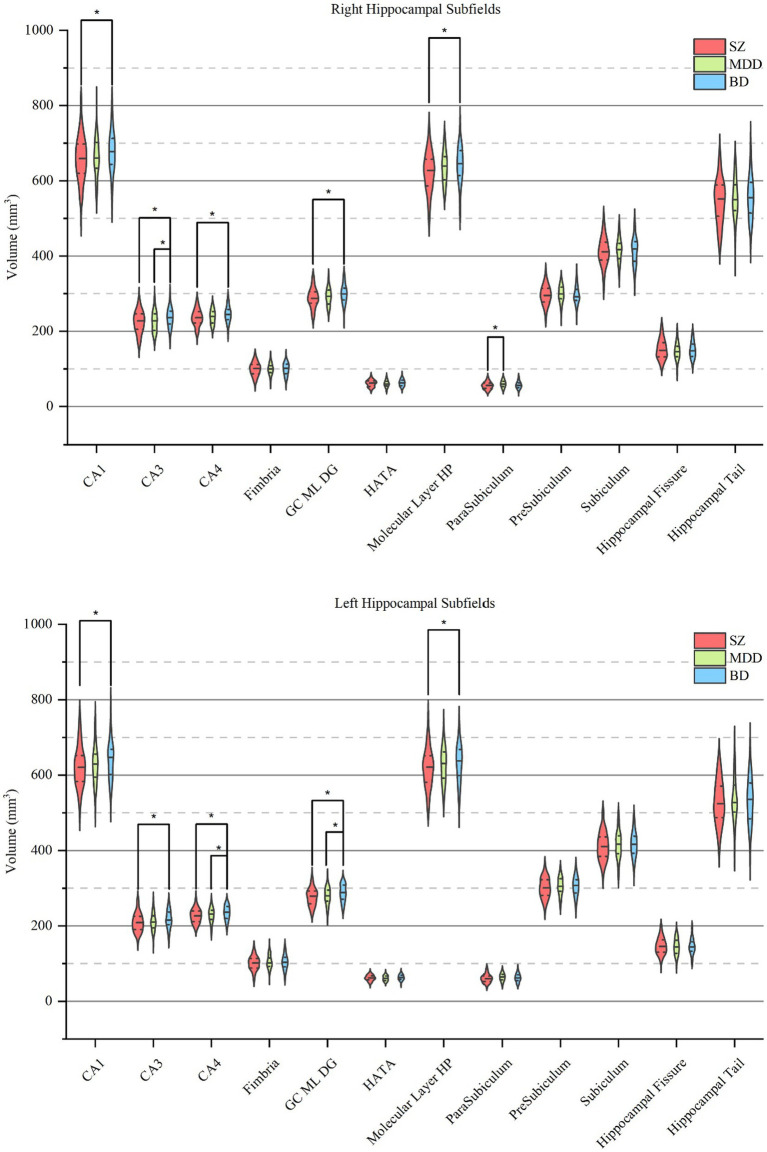
Pairwise comparisons of hippocampal subfield volumes across SZ, MDD, and BD. Each violin plot shows the volume distribution of each hippocampal subfield, in which a solid line and two dotted lines indicate the median and quartiles, respectively. Pairwise volume differences were significant at the 0.05 (∗) level after false discovery rate correction at the subfield level. SZ, patients with schizophrenia; MDD, patients with major depression disorder; BD, patients with bipolar disorder; CA, cornu ammonis; GC-ML-DG, granule cells in the molecular layer of the dentate gyrus; HATA, hippocampal-amygdaloid-transition-area.

First, we investigated the diagnostic differences in the total hippocampal and hippocampal subfield volumes between SZ, MDD, and BD (Table 2) and found significant volume differences in CA1 (right, adjusted *p* = 1.15 × 10^−2^; left, adjusted *p* = 1.63 × 10^−2^), CA3 (right, adjusted p = 1.63 × 10^−3^; left, adjusted *p* = 1.25 × 10^−2^), CA4 (right, adjusted *p* = 1.48 × 10^−3^; left, adjusted *p* = 8.99 × 10^−4^), GC-ML-DG (right, adjusted *p* = 6.39 × 10^−4^; left, adjusted *p* = 5.27 × 10^−4^), ML (right, adjusted *p* = 3.08 × 10^−3^; left, adjusted *p* = 4.67 × 10^−2^), and the whole hippocampus (right, adjusted *p* = 6.60 × 10^−3^; left, adjusted *p* = 1.07 × 10^−2^), bilaterally, and parasubiculum (adjusted *p* = 3.38 × 10^−4^) in the right hemisphere, and the total hippocampus (adjusted *p* = 4.15 × 10^−3^) across the diagnostic groups (Figure 2). *Post hoc* analysis showed that right parasubiculum of SZ was smaller than that of MDD (adjusted *p* < 0.05). Bilateral CA1, CA3, CA4, GC-ML-DG, ML and the whole hippocampus, and the total hippocampus volumes were decreased in SZ compared to BD (adjusted p < 0.05). Volume differences between MDD and BD showed that the left CA4, GC-ML-DG and right CA3 were larger in BD than in MDD (adjusted *p* < 0.05). Contrary to our hypothesis, the results showed a gradual increase in hippocampal subfield volumes from SZ to MDD to BD.

**Table 2:** Volumes of the hippocampal subfields in each diagnostic group.

		SZ	MDD	BD	Multivariate analysis of covariates		*post hoc* tests		
		(*n* = 204)	(*n* = 98)	(*n* = 110)	Diagnosis × subfield × hemisphere		SZ vs. MDD	SZ vs. BD	MDD vs. BD
		Mean ± SD	Mean ± SD	Mean ± SD	*F*_2, 405_	*p*	adjusted *p*	adjusted *p*	adjusted *p*
Whole hippocampus	T	6917.42 ± 441.78	7005.64 ± 438.81	7092.02 ± 438.67	5.56	**4.15 × 10** ^**−3**^	0.33	**3.08 × 10** ^**−3**^	0.46
	R	3501.91 ± 239.74	3547.19 ± 238.12	3592.59 ± 238.05	5.08	**6.60 × 10** ^**−3**^	0.39	**5.00 × 10** ^**−3**^	0.50
	L	3415.51 ± 233.94	3458.44 ± 232.36	3499.43 ± 232.29	4.59	**1.07 × 10** ^**−2**^	0.42	**8.54 × 10** ^**−3**^	0.61
CA1	R	658.48 ± 58.45	662.94 ± 58.05	679.16 ± 58.03	4.52	**1.15 × 10** ^**−2**^	1.00	**9.72 × 10** ^**−3**^	0.13
	L	621.87 ± 54.13	627.06 ± 53.76	640.42 ± 53.75	4.16	**1.63 × 10** ^**−2**^	1.00	**1.30 × 10** ^**−2**^	0.22
CA3	R	224.65 ± 29.09	225.52 ± 28.90	236.61 ± 28.88	6.52	**1.63 × 10** ^**−3**^	1.00	**1.92 × 10** ^**−3**^	**1.70 × 10** ^**−2**^
	L	209.09 ± 25.37	210.76 ± 25.19	217.95 ± 25.19	4.43	**1.25 × 10** ^**−2**^	1.00	**1.11 × 10** ^**−2**^	0.12
CA4	R	236.04 ± 22.10	238.41 ± 21.95	245.56 ± 21.94	6.62	**1.48 × 10** ^**−3**^	1.00	**1.05 × 10** ^**−3**^	0.06
	L	226.64 ± 19.82	228.90 ± 19.68	235.51 ± 19.68	7.14	**8.99 × 10** ^**−4**^	1.00	**6.17 × 10** ^**−4**^	**4.60 × 10** ^**−2**^
Fimbria	R	99.25 ± 17.63	99.62 ± 17.51	99.64 ± 17.50	0.02	0.98	1.00	1.00	1.00
	L	101.06 ± 19.37	104.94 ± 19.23	104.16 ± 19.22	1.63	0.20	0.32	0.54	1.00
GC-ML-DG	R	288.28 ± 26.12	291.99 ± 25.94	300.33 ± 25.94	7.49	**6.39 × 10** ^**−4**^	0.76	**3.97 × 10** ^**−4**^	0.06
	L	276.60 ± 24.17	279.83 ± 24.01	287.87 ± 24.00	7.69	**5.27 × 10** ^**−4**^	0.85	**3.34 × 10** ^**−4**^	**4.67 × 10** ^**−2**^
HATA	R	61.45 ± 9.33	60.44 ± 9.27	62.84 ± 9.26	1.81	0.17	1.00	0.63	0.18
	L	61.94 ± 7.91	61.28 ± 7.87	63.38 ± 7.87	2.04	0.13	1.00	0.38	0.16
Molecular layer	R	623.42 ± 50.63	636.88 ± 50.29	643.23 ± 50.28	5.87	**3.08 × 10** ^**−3**^	0.10	**3.46 × 10** ^**−3**^	1.00
	L	617.15 ± 51.78	626.32 ± 51.42	632.10 ± 51.40	3.09	**4.67 × 10** ^**−2**^	0.46	**4.83 × 10** ^**−2**^	1.00
Parasubiculum	R	55.44 ± 9.93	60.41 ± 9.86	57.37 ± 9.86	8.15	**3.38 × 10** ^**−4**^	**1.96 × 10** ^**−4**^	0.31	0.08
	L	60.69 ± 11.34	64.01 ± 11.27	62.48 ± 11.26	2.90	0.06	0.06	0.56	0.98
Presubiculum	R	296.63 ± 25.31	299.58 ± 25.13	296.70 ± 25.13	0.50	0.61	1.00	1.00	1.00
	L	300.85 ± 26.84	306.21 ± 26.66	305.28 ± 26.65	1.66	0.19	0.33	0.50	1.00
Subiculum	R	412.33 ± 37.15	417.03 ± 36.90	414.17 ± 36.89	0.52	0.59	0.92	1.00	1.00
	L	411.60 ± 37.15	415.57 ± 36.90	417.02 ± 36.89	0.84	0.43	1.00	0.67	1.00
Hippocampal fissure	R	152.55 ± 25.24	147.40 ± 25.07	150.73 ± 25.06	1.35	0.26	0.30	1.00	1.00
	L	146.85 ± 22.31	145.08 ± 22.16	144.97 ± 22.15	0.33	0.72	1.00	1.00	1.00
Hippocampal tail	R	545.95 ± 58.37	554.38 ± 57.98	556.98 ± 57.97	1.45	0.24	0.74	0.34	1.00
	L	528.02 ± 60.89	533.57 ± 60.48	533.26 ± 60.46	0.39	0.68	1.00	1.00	1.00

Bonferroni correction was applied: adjusted *p* = *p* × 26.

SZ, patients with schizophrenia; MDD, patients with major depression disorder; BD, patients with bipolar disorder; CA, cornu ammonis; GC-ML-DG, granule cells in the molecular layer of the dentate gyrus; HATA, hippocampal-amygdaloid-transition-area; T, total; R, right; L, left. Absolute volume means ± SDs are shown. *p* values are shown in boldface if *p* < 0.05 (adjusted for multiple comparisons), and *post hoc* analysis was performed.

### Volume differences of hippocampal subfields in different subgroups

Next, we examined the differences in subfield volumes between participants with and without medication treatment. In SZ group, we found significantly larger volumes in left HATA (adjusted *p* = 3.62 × 10^−2^) and parasubiculum (adjusted *p* = 3.67 × 10^−2^) in antipsychotics-naïve patients. Among the three groups, MDD group had the most subfields with differences. Never-treated patients had smaller subfield volumes in right CA1 (adjusted *p* = 3.71 × 10^−2^), CA4 (adjusted *p* = 5.97 × 10^−3^), ML (adjusted *p* = 2.12 × 10^−4^), subiculum (adjusted *p* = 3.35 × 10^−3^) and whole hippocampus (adjusted *p* = 2.13 × 10^−3^), left fimbria (adjusted *p* = 2.91 × 10^−2^), bilateral GC-ML-DG (right, adjusted *p* = 5.18 × 10^−3^; left, adjusted *p* = 3.87 × 10^−2^), and the total hippocampus (adjusted *p* = 1.10 × 10^−2^). In BD group, never-treated patients had smaller subfield volumes in right HATA (adjusted *p* = 3.97 × 10^−2^) and fissure (adjusted *p* = 4.28 × 10^−2^), and left presubiculum (adjusted *p* = 1.00 × 10^−2^). A MANCOVA with age, gender, education, and ICV as covariates was performed between the SZ, MDD, BD-I, and BD-II. We found that differences in hippocampal subfield volumes between BD-I, SZ, and MDD contributed to all the differences between BD and other diagnoses in the main analysis. There were no significant subfield volume differences between BD-I and BD-II, SZ and BD-II, or MDD and BD-II. Moreover, there were no significant differences in subfield volumes between lithium-treated (*n* = 63) and non-treated (*n* = 47) patients with BD using the same statistical method. Detailed information on hippocampal subfield volumes in each subgroup is described in Supplementary Tables S1–S3.

### Correlations with clinical characteristics

**Figure 3:**
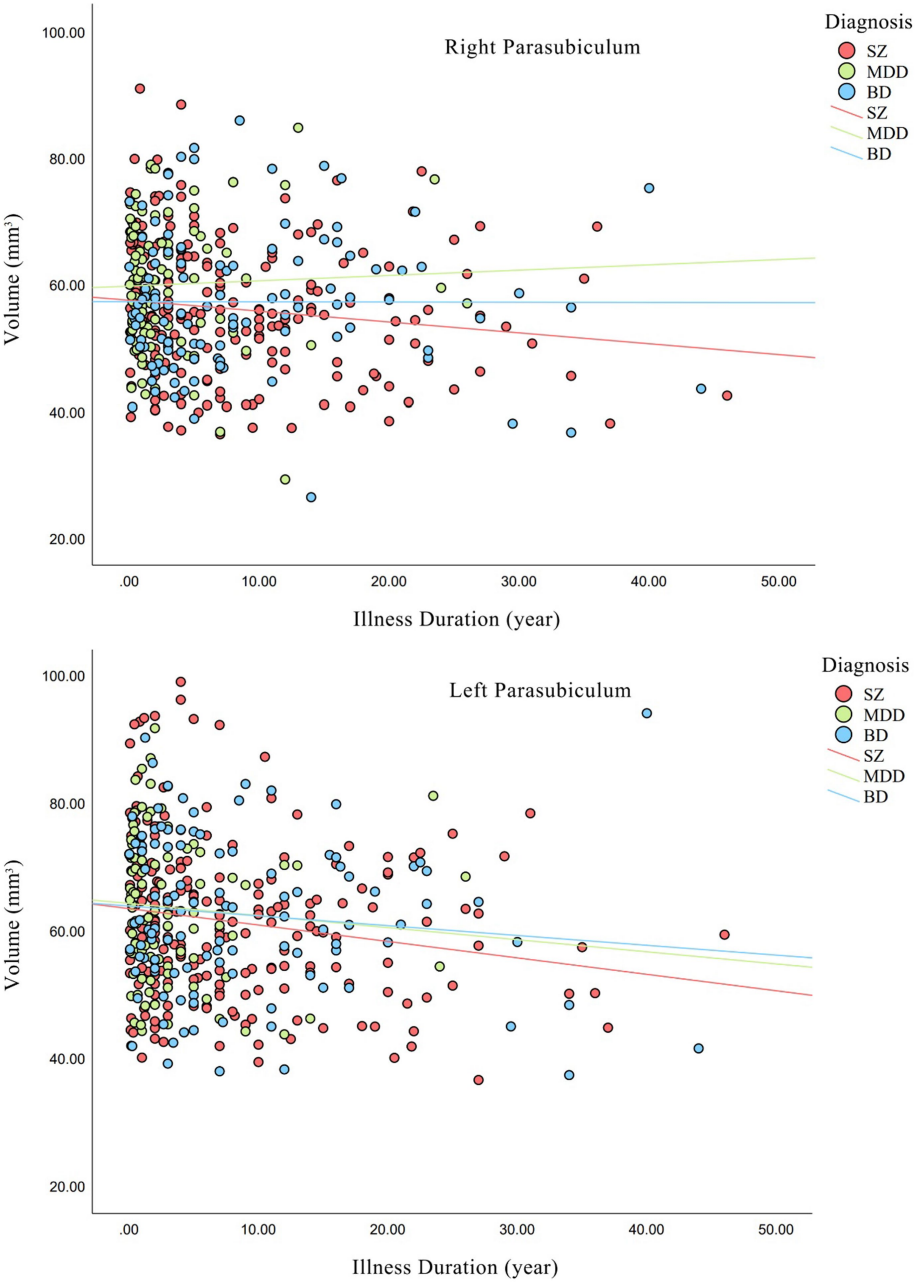
Relationships between illness duration and hippocampal volumes in each diagnostic group. Non-parametric correlations were statistically significant for bilateral parasubiculum (right, *p* = 2.52 × 10^−2^; left, *p* = 1.35 × 10^−2^) in SZ group. SZ, patients with schizophrenia; MDD, patients with major depression disorder; BD, patients with bipolar disorder.

We investigated whether the hippocampal subfield volumes were related to illness duration and drug dose. As shown in Figure 3, a negative correlations was found in bilateral parasubiculum (right, Kendall’s tau = −0.11, *p* = 2.52 × 10^−2^; left, Kendall’s tau = −0.12, *p* = 1.35 × 10^−2^) in SZ group. Meanwhile, no significant correlation was found between illness duration and hippocampal subfield volumes in MDD or BD groups. As for the correlations between drug dose and subfield volumes, we found that in SZ group, chlorpromazine equivalents and hippocampal subfield volumes in bilateral subiculum (right, Kendall’s tau-b = 0.11, *p* = 3.67 × 10^−2^; left, Kendall’s tau-b = 0.12, *p* = 2.96 × 10^−2^) and right presubiculum (Kendall’s tau-b = 0.11, *p* = 3.54 × 10^−2^) had a significant positive correlation. There was a positive correlation between fluoxetine equivalents and volume of right CA4 (Kendall’s tau-b = 0.21, *p* = 1.26 × 10^−2^) and GC-ML-DG (Kendall’s tau-b = 0.21, *p* = 1.18 × 10^−2^) in MDD, but no significant correlation between lithium and any subfields in BD. As for HAMD factors and hippocampal subfield volumes, positive correlations were found between right CA1 and anxiety/somatization and diurnal variation, left subiculum and weight, and left tail and diurnal variation. Negative correlations were found in left CA3, CA4, and GC-ML-DG and cognitive impairment and sleep disorders, left HATA and retardation, left CA3, GC-ML-DG, and total HAMD scores. Detailed results are shown in Supplementary Tables S4, S5.

### Laterality differences in hippocampal subfield volumes between and within SZ, MDD, and BD

No significant differences were found in the laterality of hippocampal subfield volumes between SZ, MDD, and BD. Within each group, all groups were found to have smaller left whole hippocampus, CA1, CA3, CA4, GC-ML-DG, ML and hippocampal tail, while larger left parasubiculum and presubiculum. In addition, the left hippocampal fissure was smaller in SZ and BD, whereas the left fimbria was larger in MDD and BD (Supplementary Tables S6, S7).

## Discussion

The present study investigated whether the anatomy of hippocampal subfields changes in the continuum of the three major psychiatric disorders and found similar changes across the three disorders, but also some notable differences. We found a relationship in which hippocampal subfield volume reduction varied with psychotic diagnosis, with severity increasing from BD to MDD to SZ (contrary to hypothesis). The trends of all subfields were identical, suggesting similar hippocampal pathophysiologic progress in the three disorders. On the other hand, subgroup analysis of patients with and without medication treatment showed there were possible factors associated with changes in hippocampal subfield volumes. More importantly, comparisons of transdiagnostic discrepancies revealed patterns of disease development, indicating that the three disorders may have grown from the same continuum. These findings suggested that the hippocampus could influence the progression of psychiatric disorders via direct or indirect mechanisms. SZ, MDD, and BD may be in a specific continuum at the hippocampal level.

Regarding the total hippocampus volumes, our finding was consistent with a meta-analysis that reduced hippocampal volumes may be a common feature across the psychosis continuum, and volume loss is greater in SZ compared to BD (53). Furthermore, a recent research found that SZ has smaller bilateral hippocampus than BD, and their ROC analysis showed that right hippocampal volume is a potential marker to distinguish SZ from BD (54). A previous study comparing hippocampal subfields between BD and MDD found no significant difference between the two groups, which is consistent with our findings, as we also found no differences in the total and bilateral whole hippocampus between MDD and BD groups (41). However, another similar study found that BD was associated with lower volumes of bilateral whole hippocampus, while a meta-analysis showed the opposite conclusion that MDD was associated with significantly smaller hippocampus than BD (55, 56). As for SZ and MDD, a study on hippocampal volumes in SZ, MDD, and HC showed significantly reduced hippocampal volumes in MDD compared to SZ (45). Additionally, a study including SZ, MDD, BD, and HC compared the differences in subcortical brain volumes and found larger right hippocampus in MDD than in SZ and BD, but no significant differences between SZ and BD (22). Based on these results, it can be inferred that the hippocampus in SZ is smaller than that of BD; however, the status of hippocampus in MDD remains puzzling, which also appears in the relationship of hippocampal subfields across the three disorders.

In the present study, we found 12 significant volume differences between SZ and BD, contributing to most of the discrepancies in the three groups. Our findings are supported by a previous meta-analysis, which reported that volume reductions in left CA1, CA2/3, and CA4/DG were more pronounced in SZ than in BD (44). These subfields are located on the DG-CA3-CA1 pathway, the trisynaptic circuitry (57). Hippocampal alterations are thought to be synaptic or dendritic lesions that are mediated by developmentally disruptions in glutamatergic excitatory and/or inhibitory interneurons (58–60). Several studies have reported the role of glutamate in both SZ and BD (61, 62). A study focusing on hippocampal glutamate and volume deficits suggested that the reduced hippocampal volumes in SZ was associated with an increase in glutamatergic metabolites (63). Another study found that glutamatergic hyperactivity initiated in CA1 may be caused by a lack of inhibition of glutamatergic input from CA3, followed by dopaminergic hyperfunction, which predicts the occurrence of hippocampal atrophy during progression of psychosis, suggesting glutamate as a pathogenic driver of psychotic disorders (64). Indeed, CA1 has more expression of NMDA receptors and is especially vulnerable to glutamate-mediated neurotoxicity (65, 66). Extracellular glutamate that first accumulates in CA1 hypermetabolism, affecting metabolism and blood flow, leading to excitotoxic damages and secondary CA1 and CA2/3 volume loss (63, 67, 68). Given the subfields in the trisynaptic circuitry, including CA3, CA4, and GC-ML-DG, pathologic alterations gradually spread to neighboring subfields, interfering with their connections. Although studies on hippocampal glutamate in BD are limited, disorders in hippocampal glutamate may partly participate in the common reduction in subfield volumes in the trisynaptic circuitry across the psychosis continuum. The research of ML volume using sMRI is novel because ML was rarely labeled in former studies. The ML consists of the interneurons synaptic connections between CA and subiculum and works in the regulation of inner activities of the hippocampus (69). However, the pathological role of ML remains unclear in SZ and BD and the volume decrease may indicate a loss of pyramidal cells and interneurons, which may be involved in the progression of these disorders.

Differences between SZ and MDD were only found in the right parasubiculum. A recent network mate-analysis also found smaller bilateral parasubiculum in SZ than in MDD (70). The parasubiculum is originally and functionally parahippocampal and has an important role as the input hub of the entorhinal cortex (71). It is involved in scene-based cognitive and spatial processing, which was found to be significantly impaired in SZ compared to MDD (72, 73). The parasubiculum is also associated with the integration of information procession between hippocampus and cortex. The structural connections between the hippocampus and cortical/subcortical areas relates to cognitive impairment have been found in SZ but not in MDD (74, 75). Two recent studies reported a volume reduction in parasubiculum in patients with MDD compared to HC, which correlated with plasma BDNF levels (76, 77). The neurotrophin hypothesis of depression suggests that depression or stress can decrease BDNF levels and induce a reduction in cells in the hippocampus, and that antidepressants may inhibit depression-like behavior by increasing BDNF expression in patients with MDD, thus reversing the above pathological progression (78, 79). The BDNF hypothesis could potentially explain our results that antidepressant treatment partly reversed the atrophy of parasubiculum in MDD. However, these findings should be interpreted with caution and require future studies. In addition, previous studies have rarely labeled the parasubiculum, and we suggest that future work should adopt updated segmentation tools to include this subfield.

Between BD and MDD, our comparison showed significantly smaller right CA3 and left CA4 and GC-ML-DG in MDD group. Cao’s study also found difference in CA4 and GCL between the two groups, but in the left hemisphere, which is in line with the preclinical research that chronic stress and glucocorticoid exposure result in the death of CA3 pyramidal cells, induce dendritic retraction, suppress neurogenesis and reduction in granule cells in DG, finally leading to the volume loss in CA3 and GC-ML-DG (55, 80–83). Another possible mechanism of atrophy in DG may be associated with the inhibitory effects on the function of the hypothalamic–pituitary–adrenal axis (HPA). DG is involved in a negative feedback function that terminates the stress response and is regulated by stress hormones produced in the adrenal gland. It is distributed with the highest density of receptors for stress hormones, making it the most stress-sensitive structure in the brain and dependent on hormones to live (84, 85). However, persistent and chronic stress can cause neuronal structural modification and dysfunction of the DG, ultimately bringing about structure atrophy. CA4 is an important anatomical site for innervation pathways, which provides a vital link between the hippocampus and other brain regions, and loss of CA4 may impede these connections. Considering that CA4 lies between the hilum of the DG and CA3 and is a functional part of DG, we inferred that CA4 has the same pathological changes as CA3 and DG, and atrophy occurs in patients with MDD (86).

However, previous studies on hippocampal volumes between MDD and BD have shown different patterns. Three studies reported no volume differences between the two groups, whereas Cao et al. (55) found differences in CA4 and GCL in BD group, but with smaller volumes (41, 43, 55, 87). Given the earlier onset age, longer illness duration, and greater number of episode times in BD group compared with MDD group in the present study, the greater loss of hippocampal subfield volumes in MDD was indeed unexpected. To further explore the reason for these contrary results, we analyzed the correlations between hippocampal subfield volumes and HAMD factors in MDD. According to the different subfields between MDD and BD in the main analysis, we primarily focused on the correlations of CA3, CA4, and GC-ML-DG. The results were surprisingly consistent with the main analysis, showing that all left CA3, CA4, and GC-ML-DG were negatively correlated with cognitive impairment and sleep disorders, while left CA3 and GC-ML-DG were also negatively correlated with total HAMD scores (Supplementary Table S8). HAMD is an important measure of the severity of depressive states and has been found to be negatively correlated with hippocampal volumes in MDD in previous studies, with a similar correlation for right DG (88, 89). Among the symptoms of MDD, sleep disorders is a prominent symptom and a frequently reported item in HAMD assessment, while cognitive impairment is a core feature of depression secondary to emotional symptoms (90, 91). The results showed that the severity of clinical symptoms of MDD, especially cognitive impairment and sleep disorders, had a significant impact on the volume reduction in CA3, CA4, and GC-ML-DG. It is possible that such volume reduction is related to clinical symptoms of MDD, resulting in the smaller hippocampal subfields. On the other hand, deficits in hippocampal subfields present in the acute first episode of MDD, number the granule cells and glial cells, and density of pyramidal neurons in CA1 increased with the duration of depressive illness in recurrent/chronic MDD (92, 93). Longitudinal studies have shown that hippocampal abnormalities in MDD are reversible with remission of symptoms, with greater increases in hippocampal volumes in patients with MDD in remission compared to HC (94–96). Similarly, alterations in hippocampal subfields have been found to be affected by long-term lithium treatment. Lithium prevents and reverses stress-induced dendritic atrophy of hippocampal principal cells, leading to an increase in hippocampal subfield volumes (97–100). The evidence provided by Palmos et al. (52) suggests that chronic lithium treatment increased the differentiation of human hippocampal progenitor cells, followed by an increase in the volume of GC-ML-DG and then the total hippocampus (52). Furthermore, our results are supported by the meta-analysis that the hippocampal volume is smaller in patients with MDD than in BD, and the illness duration is shorter in patients with MDD than in BD (56, 101). In summary, we supposed that in the present study, the high proportion of acute episodes and short illness duration in patients with MDD and the long-term lithium exposure in patients with BD in the present study were responsible for the lower hippocampal subfield volumes in MDD compared with BD. The illness duration of SZ and BD was nearly matched, and both groups showed exact hippocampal differences, as we hypothesized. Such mismatch in the illness duration of MDD may also be the reason for its ambiguous relationship in the continuum. Future studies should control for illness duration, as this could better reveal the hippocampal alterations between the three disorders, especially between MDD and BD.

Comparing of hippocampal subfield volumes between patients with and without medication treatment showed several differences between the three groups. In SZ, antipsychotics-naïve patients showed larger left HATA and parasubiculum than those with medication. In addition, we found a positive correlation between chlorpromazine equivalents and volumes of right presubiculum and bilateral subiculum. Parasubiculum is innervated by projections from the subiculum and CA1 (102). The subiculum, presubiculum and parasubiculum form the human subicular complex, where information flows from the hippocampus to the entorhinal cortex (71, 103). An increase in the volume of subiculum has been reported in patients with psychosis after antipsychotic treatment (104). The subicular complex is considered to be one of the site of action of antipsychotics (105). HATA is the hippocampus-amygdala transition area that participates in the connection between the hippocampus and amygdala. There were few studies on HATA in SZ. A review found that the effects of antipsychotic treatment on hippocampal subfields in SZ may be distinct, as acute treatment reduces subfield volumes, whereas long-term treatment is beneficial (106). Considering that some of our participants were in an acute antipsychotic treatment period, their hippocampal subfield volumes changes may explain the fact that antipsychotic treatment has a pharmacological effect on HATA. However, due to the presence of several confounding factors, future studies on antipsychotics effects on the hippocampus are needed.

In the present study, never-treated patients with MDD had smaller subfield volumes in the right CA1, CA4, ML, subiculum, and whole hippocampus, left fimbria, bilateral GC-ML-DG, and the total hippocampus. Correlation analysis showed a positive correlation between fluoxetine equivalents and hippocampal volumes in right CA4 and GC-ML-DG. Huang et al. (107) found that DG volumes were lower in unmedicated MDD compared with medicated MDD. As we discussed above, chronic stress leads to volume loss in CA1, CA4, and GC-ML-DG, and antidepressant treatment prevents or reverses the pathological changes in these subfields and increases their volumes (83). The subiculum is likely a primary mode of hippocampal interaction with the HPA axis (81, 108, 109). We suppose that the effects of antidepressant treatment acts not only on the reduction in CA and DG, but are also participates in neuroplastic changes in the subiculum (107). ML covers CA and DG and consists of inter-regional synapses between subfields, while fimbria is an important white matter relay connecting the hippocampus to the paraventricular nucleus of the hypothalamus and other limbic regions (110). The CA1, subiculum, and ML are part of the hippocampal connectivity pathway, where interneurons bridging CA and the subiculum form ML and subiculum receives inputs from pyramidal neurons of CA1 (111). The atrophy of all three subfields in unmedicated patients with MDD suggested that disruption of hippocampal connections may contribute to pathologies in MDD, and that antidepressant may mitigate or even reverse such effect and halt the progression of MDD.

In BD group, we found that BD-I contributed to all the differences with other diagnoses. Cao et al. (55) found that reduction in subfield volumes was more prominent in BD-I than in BD-II, but compared with HC. They concluded that the hippocampus is more altered in the pathophysiology of BD-I than in BD-II. However, no significant differences showed in the direct comparison between BD-I and BD-II, which may be due to the smaller proportion of individuals with BD-II. The greater clinical heterogeneity in BD-II compared to BD-I may also have confounded the results (112).

Compared with patients who had received medication prior to the study, never-treated patients had smaller subfield volumes in the right HATA, left presubiculum and hippocampal fissure. Just like the effects of antidepressants on the trisynaptic circuit, mood stabilizers induce changes in hippocampal strength and synaptic plasticity. Exposure to stress and excess glucocorticoids leads to dendritic retraction and induction of apoptotic cell signaling (84). Lithium treatment prevents and reverses stress-induced hippocampal dendritic atrophy in hippocampal principal cells and increases the hippocampal subfield volumes (97–100). Simonetti compared hippocampal subfield volumes between HC and BD patients with short-term and long-term lithium treatment and found that left presubiculum and right subiculum were larger in BD patients exposed to lithium for more than 24 months (113). Furthermore, a recent review found that total hippocampal and hippocampal subfield volumes were larger in BD patients with lithium treatment than in those without, thus suggesting that lithium may be associated with increased hippocampal volumes in BD (114). However, some of our drug-naïve patients were in their chronic course and it is not possible to completely exclude their hippocampal volumes changes related to illness duration, which should be taken into consideration.

To further control the effects of current lithium use, we conducted a subgroup analysis of lithium-treated and non-treated patients with BD and found no significant subfield volumes differences. Although the result is consistent with the findings of Haukvik et al. (115), it needs to be interpreted with caution. Wise et al. (101) performed a meta-regression analysis with lithium as a moderator and found that lithium use could not explain the between-study heterogeneity. This study may indicate that lithium is not correlated with hippocampal volume. Our results, together with the lack of an association of illness duration, antipsychotics, or lithium on any of the subfield volumes, may suggest that effects of current lithium use were not sufficient to affect hippocampal subfield volumes. In general, considering the heterogenicity of previous studies and findings, future studies may further discuss the effect of BD type and lithium use (pre- and intra-study phases) on hippocampal subfields in patients with BD.

In the secondary analysis, we found a significant negative correlation between illness duration and volumes of bilateral parasubiculum in SZ, but no correlation in MDD or BD groups. These findings must be interpreted with caution because the existing correlations were weak and unbalanced distribution of illness duration decreased the strength of the association below statistical significance. Ho concluded that illness duration was negatively associated with CA1 volumes in SZ (40). Another study reported that volumes of left CA1, CA4, ML, presubiculum and subiculum were inversely correlated with illness duration in BD, while no significant correlation was found in MDD (41). We also tested the effects of medication use on hippocampal subfield volumes, observing weak relationships between chlorpromazine equivalents and volumes of bilateral subiculum and right presubiculum in SZ, fluoxetine equivalents and volumes of right CA4 and GC-ML-DG in MDD, and no relationship between lithium and volumes of any subfield in BD. We exercise caution on these correlations, as our participants were prescribed multiple medications with varying durations and doses. Many studies have shown that hippocampal subfield volumes correlate with various clinical and biological factors, including illness duration, onset age, remission states, and severity of symptoms (41, 45, 56, 116). Additionally, the number of prior episodes may have been a confounding factor in our analysis, as hippocampal subfield volumes decrease with increasing episode times (55, 117, 118). It was difficult to separate disease from affective episodes, medication effects, and onset age, thus our ability to rule out the effects of confounding factors on our hypothesis is less definitive. Further research should analyze both first-episode and chronic patients, and longitudinal studies focusing on illness duration are necessary to better explain this issue.

Finally, we analyzed the laterality differences in hippocampal subfield volumes between and within the three groups, and found an extraordinary consistency in that7 hippocampal subfields were smaller in the left hemisphere and only 2 were larger, within each group. In addition, no significant difference in laterality was found in any of the 13 subfields between diagnostic groups. It has been shown that left hippocampus is smaller in patients with SZ, while the human hippocampus demonstrates a dominant volume in the right hemisphere (119). The same pattern of hippocampal asymmetry has been found in MDD (120, 121). Only a few studies have focused on asymmetric changes in hippocampal subfields in BD, although Javadapour conducted a correlation analysis and found larger hippocampus on the right side than on the left side, albeit not statistically significant (122). Normal brain development proceeds in a sequence that growth of temporal and occipital structures completes relatively late, and the left hippocampus completes its development more slowly than the right, making these structures more vulnerable to developmental and prenatal stressors (123, 124). Accordingly, these alterations in asymmetry are presumed to be related to the etiology of SZ, and the disease process interferes in some ways with the mechanisms of lateralization (125, 126). A so-called ‘right-shift factor’ is associated with right-handedness, left cerebral dominance for language, and normal cerebral asymmetry, which may be a locus where genetic aberrations predispose for SZ, which may be the result of genetic abnormalities in humans (127–129). However, existing studies on asymmetry in psychoses are contradictory, and the significance of asymmetry in hippocampal subfields remains unclear. Based on the similarity of asymmetry in hippocampal subfields in this study, we suggest that the three disorders overlap in terms of pathobiology, etiology, and disease progression. More in-depth studies are needed to reveal the asymmetry patterns and the underlying mechanisms of the three disorders.

In the present study, we investigated the differences in hippocampal subfield volumes between SZ, MDD and BD. Indeed, according to previous studies, schizophrenia and affective disorders share common symptoms, neurotransmitter dysfunction, treatment strategies, genetic transmissibility, and risk factors (12, 17, 130–132). Accordingly, it becomes an emerging consensus to comprehend the three disorders along a dimensional continuum, rather than distinct categorical diagnoses. Our study found continuous changes in volumes at the level of hippocampal inner phenotype. The pattern of atrophy of hippocampal subfields is most severe in SZ, moderate in MDD, and mildest in BD, suggesting a gradient in pathological progression across the three disorders, with SZ being the worst along the specific continuum. The distinct alterations in the subfields reflect the different origins of the lesions in this continuum. It is clear that CA1 performs the trigger point of hippocampal atrophy in SZ and extends to the rest part of the hippocampus along the trisynaptic pathway (133). Although the mechanism of hippocampal atrophy in MDD and BD remains dubious, considering the very close subfield volumes between MDD and SZ, we assume that the hippocampal lesions in MDD follow the same pathway as SZ but spread more slowly and reach the parasubiculum later. As for BD, we believe that the alterations of hippocampal subfield volumes are also centered in CA and DG core functional area and spread throughout the entire hippocampus; therefore, SZ, MDD, and BD may be in a continuum and share the same pathological mechanisms in the hippocampus, but display different clinical symptoms due to distinct progression. Besides, temporal continuity is another nonnegligible factor. Psychotic experiences at a subclinical level in adolescence and early adulthood are valuable in predicting psychosis, functional impairment, violence, and suicide (134). Future research should combine cross-sectional and longitudinal studies of hippocampal subfield volumes for the three disorders.

### Study limitation

A major limitation of the present study is that we did not include healthy individuals as a comparison group. Although most previous studies have shown atrophy of hippocampal subfields in major psychiatric disorders compared to healthy individuals, having a control group in our study could better explain the pathological patterns and progression of the continuum. Another limitation is that we did not assess participants with identical clinical scales, so further statistical analysis was not possible. Scales are important phenotypes that reflect the different pathophysiology shared across psychoses (135). Numerous studies have found a correlation between hippocampal subfield volumes and psychotic symptoms (136, 137). Besides, cognitive impairment, a core symptom of psychosis, has also been correlated with hippocampal subregion volume (133, 138). We did not collect sufficient data on cognitive functions, which restricted the analysis of the correlation between hippocampal subfields malfunction in the three disorders. Future investigations should explore the association with ample clinical scale data. Additionally, our *post-hoc* analyses have evaluated the influences of BD type and lithium on hippocampal subfield volumes, but we were unable to fully control for their effects in the present study. Also, some of our participants were taking antiepileptics or sedative-hypnotic drugs, and we could not completely exclude their effects on the hippocampus (139, 140). Such confounding factors and others, such as previous episode times, non-pharmacological treatment methods and onset age should be considered in future research. Finally, as previously mentioned, illness duration may be an important factor influencing the pathological progression along the continuum, and we hope that future studies focusing on the hippocampus will take this into account.

## Conclusion

We found a gradient of volume reduction in the hippocampal subfields in SZ, MDD, and BD, with the most severe in SZ and the mildest in BD. Therefore, we propose to divide the three disorders into a specific continuum, as this would allow us to better understand the mechanisms of psychiatric disorders. Furthermore, the hippocampus is thought to be a critical phenotype to identify the continuum remains to be tested, that phenotypes definitions will lead to the identification of successful treatments using biomarkers. There are possible clinical applications from the psychosis continua and their biomarkers, such as predicting outcomes in early psychosis treatment, predicting improvements in behavioral, cognitive, or clinical features, and predicting outcomes of pharmacological interventions (141). We expect more biological evidence to support the concept of the psychosis continuum. Lastly, it is necessary to explore the pathophysiological mechanisms underlying the association between alterations in hippocampal subfields and clinical symptoms.

## Data availability statement

The raw data supporting the conclusions of this article will be made available by the authors, without undue reservation.

## Ethics statement

The studies involving human participants were reviewed and approved by Affiliated Brain Hospital of Nanjing Medical University Ethics Committee. The patients/participants provided their written informed consent to participate in this study.

## Author contributions

YS: conceptualization, writing—reviewing and editing, supervision, and project administration. PC: methodology, data curation, software, formal analysis, writing—original draft preparation, writing—reviewing and editing, and visualization preparation. CC, QS, and YL: validation. FR, CH, JZ, XW, and GX: investigation and resources. All authors contributed to the article and approved the submitted version.

## Funding

This work was supported by Scientific Research Foundation of Nanjing Medical University (201715048 and ZKX17030).

## Conflict of interest

The authors declare that the research was conducted in the absence of any commercial or financial relationships that could be construed as a potential conflict of interest.

## Publisher’s note

All claims expressed in this article are solely those of the authors and do not necessarily represent those of their affiliated organizations, or those of the publisher, the editors and the reviewers. Any product that may be evaluated in this article, or claim that may be made by its manufacturer, is not guaranteed or endorsed by the publisher.
