# Supplementary material for: Volumes of hippocampal subfields suggest a continuum between schizophrenia, major depressive disorder and bipolar disorder
Source: Front Psychiatry. 2023 Jul 20;14:1191170. doi: 10.3389/fpsyt.2023.1191170 (PMC10400724; doi:10.3389/fpsyt.2023.1191170)
Supplement: Supplementary file 1 [file Data_Sheet_1.docx]

Table S1

The differences in the volumes of hippocampal subfields between patients with and without medication treatment in SZ, MDD and BD groups.

|  |  | SZ | | | | MDD | | | | BD | | | |
| --- | --- | --- | --- | --- | --- | --- | --- | --- | --- | --- | --- | --- | --- |
|  |  | With  (n=160) | Without  (n=44) |  |  | With  (n=69) | Without  (29) |  |  | With  (n=98) | Without  (12) |  |  |
|  |  | Mean ± SD | Mean ± SD | *F_1,198_* | adjusted *P* | Mean ± SD | Mean ± SD | *F_1,92_* | adjusted *P* | Mean ± SD | Mean ± SD | *F_1,104_* | adjusted *P* |
| Whole Hippocampus | T | 6985.46 ± 461.74 | 7004.03 ± 471.63 | 0.05 | 0.82 | 6977.85 ± 366.50 | 6764.45 ± 369.47 | 6.73 | **1.10 × 10^-2^** | 7052.74 ± 428.66 | 6930.64 ± 436.71 | 0.83 | 0.36 |
|  | R | 3533.54 ± 255.04 | 3556.65 ± 260.51 | 0.27 | 0.61 | 3541.15 ± 185.95 | 3409.26 ± 187.45 | 9.99 | **2.13 × 10^-3^** | 3575.90 ± 230.62 | 3474.49 ± 234.95 | 1.98 | 0.16 |
|  | L | 3451.92 ± 237.31 | 3447.38 ± 242.39 | 0.01 | 0.91 | 3436.69 ± 212.83 | 3355.19 ± 214.56 | 2.91 | 0.09 | 3476.84 ± 228.03 | 3456.15 ± 232.32 | 0.08 | 0.77 |
| CA1 | R | 665.43 ± 61.21 | 665.31 ± 62.52 | 0.00 | 0.99 | 661.55 ± 48.98 | 638.30 ± 49.37 | 4.48 | **3.71 × 10^-2^** | 676.94 ± 56.82 | 647.03 ± 57.89 | 2.84 | 0.10 |
|  | L | 627.33 ± 55.02 | 627.79 ± 56.20 | 0.00 | 0.96 | 624.69 ± 49.47 | 608.76 ± 49.87 | 2.06 | 0.16 | 634.96 ± 52.53 | 648.38 ± 53.51 | 0.67 | 0.42 |
| CA3 | R | 225.45 ± 30.31 | 230.48 ± 30.96 | 0.90 | 0.35 | 226.86 ± 27.02 | 215.51 ± 27.24 | 3.51 | 0.06 | 235.42 ± 25.80 | 230.67 ± 26.29 | 0.35 | 0.56 |
|  | L | 211.02 ± 25.89 | 213.14 ± 26.45 | 0.22 | 0.64 | 207.94 ± 24.03 | 207.67 ± 24.23 | 0.00 | 0.96 | 215.13 ± 24.34 | 224.17 ± 24.80 | 1.42 | 0.24 |
| CA4 | R | 237.48 ± 22.76 | 242.20 ± 23.24 | 1.40 | 0.24 | 239.06 ± 18.56 | 227.34 ± 18.71 | 7.92 | **5.97 × 10^-3^** | 244.34 ± 21.48 | 236.88 ± 21.88 | 1.24 | 0.27 |
|  | L | 229.00 ± 19.75 | 229.27 ± 20.17 | 0.01 | 0.94 | 228.20 ± 19.58 | 220.21 ± 19.74 | 3.31 | 0.07 | 233.56 ± 19.26 | 235.31 ± 19.63 | 0.08 | 0.77 |
| Fimbria | R | 100.86 ± 18.61 | 96.99 ± 19.01 | 1.41 | 0.24 | 99.83 ± 15.22 | 94.77 ± 15.34 | 2.19 | 0.14 | 100.28 ± 16.46 | 91.71 ± 16.77 | 2.78 | 0.10 |
|  | L | 102.50 ± 19.23 | 99.39 ± 19.63 | 0.85 | 0.36 | 106.25 ± 17.55 | 97.52 ± 17.70 | 4.91 | **2.91 × 10^-2^** | 104.56 ± 19.80 | 98.31 ± 20.17 | 1.02 | 0.31 |
| GC-ML-DG | R | 289.93 ± 26.77 | 295.70 ± 27.34 | 1.51 | 0.22 | 292.88 ± 21.94 | 278.78 ± 22.12 | 8.20 | **5.18 × 10^-3^** | 298.79 ± 25.64 | 290.62 ± 26.12 | 1.04 | 0.31 |
|  | L | 279.27 ± 24.34 | 279.68 ± 24.86 | 0.01 | 0.92 | 279.49 ± 23.23 | 268.56 ± 23.41 | 4.40 | **3.87 × 10^-2^** | 285.61 ± 23.33 | 288.60 ± 23.77 | 0.17 | 0.68 |
| HATA | R | 62.19 ± 9.31 | 62.00 ± 9.51 | 0.01 | 0.91 | 59.80 ± 8.96 | 59.23 ± 9.03 | 0.08 | 0.78 | 62.90 ± 8.97 | 57.06 ± 9.13 | 4.34 | **3.97 × 10^-2^** |
|  | L | 61.85 ± 8.03 | 64.82 ± 8.21 | 4.45 | **3.62 × 10^-2^** | 60.77 ± 7.15 | 60.08 ± 7.21 | 0.19 | 0.67 | 63.29 ± 7.66 | 60.75 ± 7.80 | 1.13 | 0.29 |
| Molecular Layer | R | 629.88 ± 53.56 | 629.25 ± 54.70 | 0.01 | 0.95 | 639.36 ± 38.63 | 605.91 ± 38.95 | 14.88 | **2.12 × 10^-4^** | 639.09 ± 49.62 | 630.13 ± 50.55 | 0.33 | 0.56 |
|  | L | 625.63 ± 53.06 | 616.46 ± 54.20 | 0.97 | 0.33 | 623.65 ± 46.40 | 605.52 ± 46.78 | 3.03 | 0.09 | 627.50 ± 50.71 | 624.89 ± 51.65 | 0.03 | 0.87 |
| Parasubiculum | R | 55.43 ± 10.13 | 57.50 ± 10.34 | 1.36 | 0.25 | 59.78 ± 9.52 | 59.93 ± 9.59 | 0.01 | 0.94 | 57.41 ± 9.30 | 54.28 ± 9.47 | 1.16 | 0.28 |
|  | L | 60.15 ± 11.56 | 64.42 ± 11.81 | 4.43 | **3.67 × 10^-2^** | 63.87 ± 10.37 | 62.34 ± 10.46 | 0.43 | 0.51 | 62.77 ± 10.79 | 58.39 ± 10.99 | 1.69 | 0.20 |
| Presubiculum | R | 299.57 ± 26.64 | 301.94 ± 27.21 | 0.26 | 0.61 | 297.60 ± 22.50 | 289.18 ± 22.68 | 2.78 | 0.10 | 294.89 ± 23.52 | 289.37 ± 23.96 | 0.56 | 0.45 |
|  | L | 304.40 ± 27.78 | 306.46 ± 28.38 | 0.18 | 0.67 | 303.35 ± 23.88 | 296.49 ± 24.07 | 1.64 | 0.20 | 304.45 ± 25.05 | 283.94 ± 25.51 | 6.88 | **1.00 × 10^-2^** |
| Subiculum | R | 416.38 ± 38.91 | 421.86 ± 39.74 | 0.65 | 0.42 | 416.50 ± 29.55 | 396.53 ± 29.79 | 9.07 | **3.35 × 10^-3^** | 412.13 ± 36.59 | 394.52 ± 37.27 | 2.37 | 0.13 |
|  | L | 418.99 ± 38.04 | 411.95 ± 38.85 | 1.12 | 0.29 | 412.33 ± 35.68 | 399.55 ± 35.96 | 2.55 | 0.11 | 414.52 ± 32.24 | 395.06 ± 32.85 | 3.73 | 0.06 |
| Hippocampal Fissure | R | 155.25 ± 27.22 | 152.45 ± 27.80 | 0.35 | 0.56 | 145.78 ± 22.47 | 141.84 ± 22.66 | 0.61 | 0.44 | 150.88 ± 22.05 | 136.77 ± 22.46 | 4.21 | **4.28 × 10^-2^** |
|  | L | 149.42 ± 22.63 | 147.95 ± 23.12 | 0.14 | 0.71 | 141.19 ± 22.53 | 144.69 ± 22.71 | 0.48 | 0.49 | 144.01 ± 20.26 | 137.83 ± 20.64 | 0.95 | 0.33 |
| Hippocampal Tail | R | 550.95 ± 59.31 | 553.44 ± 60.59 | 0.06 | 0.81 | 547.95 ± 53.73 | 543.78 ± 54.16 | 0.12 | 0.73 | 553.71 ± 56.90 | 552.21 ± 57.98 | 0.01 | 0.93 |
|  | L | 531.78 ± 58.82 | 534.01 ± 60.08 | 0.05 | 0.83 | 526.16 ± 59.16 | 528.50 ± 59.64 | 0.03 | 0.86 | 530.50 ± 61.76 | 538.36 ± 62.93 | 0.17 | 0.69 |

Bonferroni correction was applied: adjusted *P* = *P* × 26.

SZ, patients with schizophrenia; MDD, patients with major depression disorder; BD, patients with bipolar disorder; CA, cornu ammonis; GC-ML-DG, granule cells in the molecular layer of the dentate gyrus; HATA, hippocampal-amygdaloid-transition-area. T, total; R, right; L, left. Means ± SDs are shown. *P* values < 0.05 are shown in boldface.

Table S2

The differences in the volumes of hippocampal subfields between SZ, MDD, BD‐I, and BD‐II groups.

|  |  |  | BD‐I vs BD‐II | BD‐I vs SZ | BD‐II vs SZ | BD‐I vs MDD | BD‐II vs MDD |
| --- | --- | --- | --- | --- | --- | --- | --- |
|  |  | F | adjusted *P* | adjusted *P* | adjusted *P* | adjusted *P* | adjusted *P* |
| Whole Hippocampus | T | 3.71 | 1.000 | **0.013** | 0.503 | 1.000 | 1.000 |
|  | R | 3.38 | 1.000 | **0.016** | 0.790 | 1.000 | 1.000 |
|  | L | 3.09 | 1.000 | **0.036** | 0.512 | 1.000 | 1.000 |
| CA1 | R | 3.00 | 1.000 | **0.028** | 1.000 | 0.315 | 1.000 |
|  | L | 2.94 | 1.000 | 0.072 | 0.287 | 0.808 | 0.705 |
| CA3 | R | 4.36 | 1.000 | **0.005** | 0.978 | **0.040** | 1.000 |
|  | L | 3.72 | 0.794 | **0.008** | 1.000 | 0.094 | 1.000 |
| CA4 | R | 4.44 | 1.000 | **0.003** | 0.945 | 0.117 | 1.000 |
|  | L | 4.75 | 1.000 | **0.002** | 0.533 | 0.119 | 1.000 |
| Fimbria | R | 0.02 | 1.000 | 1.000 | 1.000 | 1.000 | 1.000 |
|  | L | 1.33 | 1.000 | 0.756 | 1.000 | 1.000 | 1.000 |
| GC-ML-DG | R | 5.10 | 1.000 | **0.001** | 1.000 | 0.102 | 1.000 |
|  | L | 5.12 | 1.000 | **0.001** | 0.492 | 0.117 | 1.000 |
| HATA | R | 1.22 | 1.000 | 1.000 | 1.000 | 0.505 | 1.000 |
|  | L | 1.81 | 1.000 | 1.000 | 0.493 | 0.815 | 0.268 |
| Molecular Layer | R | 4.16 | 1.000 | **0.005** | 1.000 | 1.000 | 1.000 |
|  | L | 2.15 | 1.000 | 0.195 | 0.636 | 1.000 | 1.000 |
| Parasubiculum | R | 5.45 | 1.000 | 0.606 | 1.000 | 0.255 | 0.899 |
|  | L | 2.39 | 1.000 | 1.000 | 0.591 | 1.000 | 1.000 |
| Presubiculum | R | 0.34 | 1.000 | 1.000 | 1.000 | 1.000 | 1.000 |
|  | L | 1.32 | 1.000 | 1.000 | 1.000 | 1.000 | 1.000 |
| Subiculum | R | 0.73 | 1.000 | 1.000 | 1.000 | 1.000 | 1.000 |
|  | L | 1.16 | 1.000 | 1.000 | 0.475 | 1.000 | 1.000 |
| Hippocampal Fissure | R | 0.93 | 1.000 | 1.000 | 1.000 | 1.000 | 1.000 |
|  | L | 0.57 | 1.000 | 1.000 | 1.000 | 1.000 | 1.000 |
| Hippocampal Tail | R | 1.11 | 1.000 | 1.000 | 1.000 | 1.000 | 1.000 |
|  | L | 0.29 | 1.000 | 1.000 | 1.000 | 1.000 | 1.000 |

Bonferroni correction was applied: adjusted *P* = *P* × 26.

BD, patients with bipolar disorder; CA, cornu ammonis; GC-ML-DG, granule cells in the molecular layer of the dentate gyrus; HATA, hippocampal-amygdaloid transition area. T, total; R, right; L, left. Means ± SD are shown. *P* values<0.05 are shown in boldface.

Table S3

The differences in the volumes of hippocampal subfields between lithium-treated and non-treated BD groups.

|  |  | lithium-treated BD  (n=63) | non-lithium-treated BD  (n=47) |  |  |
| --- | --- | --- | --- | --- | --- |
|  |  | Mean ± SD | Mean ± SD | *F* | adjusted *P* |
| Whole Hippocampus | T | 7035.63 ± 429.68 | 7044.50 ± 429.84 | 0.01 | 0.92 |
|  | R | 3564.94 ± 232.44 | 3564.69 ± 232.53 | 0.00 | 1.00 |
|  | L | 3470.69 ± 227.73 | 3479.80 ± 227.81 | 0.04 | 0.84 |
| CA1 | R | 673.08 ± 57.5 | 674.48 ± 57.52 | 0.02 | 0.90 |
|  | L | 629.51 ± 51.97 | 645.69 ± 51.99 | 2.60 | 0.11 |
| CA3 | R | 235.59 ± 25.79 | 233.99 ± 25.8 | 0.10 | 0.75 |
|  | L | 216.05 ± 24.47 | 216.20 ± 24.48 | 0.00 | 0.98 |
| CA4 | R | 242.43 ± 21.53 | 244.99 ± 21.54 | 0.38 | 0.54 |
|  | L | 234.30 ± 19.23 | 233.01 ± 19.24 | 0.12 | 0.73 |
| Fimbria | R | 99.69 ± 16.65 | 98.89 ± 16.66 | 0.06 | 0.80 |
|  | L | 102.78 ± 19.82 | 105.35 ± 19.83 | 0.45 | 0.50 |
| GC-ML-DG | R | 296.35 ± 25.66 | 299.97 ± 25.67 | 0.53 | 0.47 |
|  | L | 285.83 ± 23.31 | 286.08 ± 23.32 | 0.00 | 0.96 |
| HATA | R | 63.74 ± 8.97 | 60.28 ± 8.97 | 4.00 | 0.05 |
|  | L | 62.74 ± 7.68 | 63.38 ± 7.69 | 0.19 | 0.67 |
| Molecular Layer | R | 637.18 ± 49.61 | 639.37 ± 49.63 | 0.05 | 0.82 |
|  | L | 624.73 ± 50.54 | 630.56 ± 50.56 | 0.36 | 0.55 |
| Parasubiculum | R | 57.63 ± 9.3 | 56.32 ± 9.31 | 0.54 | 0.47 |
|  | L | 62.42 ± 10.86 | 62.12 ± 10.86 | 0.02 | 0.89 |
| Presubiculum | R | 295.19 ± 23.52 | 293.08 ± 23.53 | 0.22 | 0.64 |
|  | L | 303.38 ± 25.78 | 300.65 ± 25.79 | 0.30 | 0.58 |
| Subiculum | R | 411.62 ± 36.91 | 408.33 ± 36.92 | 0.21 | 0.65 |
|  | L | 413.25 ± 32.75 | 411.25 ± 32.76 | 0.10 | 0.75 |
| Hippocampal Fissure | R | 149.81 ± 22.44 | 148.72 ± 22.45 | 0.06 | 0.80 |
|  | L | 144.50 ± 20.28 | 141.77 ± 20.29 | 0.49 | 0.49 |
| Hippocampal Tail | R | 552.45 ± 56.81 | 555.01 ± 56.83 | 0.05 | 0.82 |
|  | L | 535.71 ± 61.5 | 525.53 ± 61.52 | 0.74 | 0.39 |

Bonferroni correction was applied: adjusted *P* = *P* × 26.

BD, patients with bipolar disorder; CA, cornu ammonis; GC-ML-DG, granule cells in the molecular layer of the dentate gyrus; HATA, hippocampal-amygdaloid transition area. T, total; R, right; L, left. Means ± SD are shown. *P* values<0.05 are shown in boldface.

Table S4

Correlations between illness duration and hippocampal subfield volumes in SZ, MDD and BD groups.

|  |  | SZ (n=204) | MDD (n=98) | BD (n=110) |
| --- | --- | --- | --- | --- |
| Whole Hippocampus | T | -0.01 | 0.05 | -0.04 |
|  | R | -0.01 | 0.06 | -0.04 |
|  | L | -0.01 | 0.02 | -0.07 |
| CA1 | R | -0.01 | 0.07 | -0.03 |
|  | L | -0.03 | 0.05 | -0.08 |
| CA3 | R | 0.01 | 0.07 | -0.01 |
|  | L | 0.04 | 0.00 | 0.00 |
| CA4 | R | -0.01 | 0.05 | 0.04 |
|  | L | 0.00 | 0.03 | -0.02 |
| Fimbria | R | -0.04 | 0.02 | -0.04 |
|  | L | -0.06 | 0.06 | -0.12 |
| GC-ML-DG | R | -0.01 | 0.06 | -0.01 |
|  | L | -0.01 | 0.05 | -0.03 |
| HATA | R | -0.02 | 0.09 | 0.04 |
|  | L | -0.04 | 0.06 | -0.05 |
| Molecular Layer | R | 0.01 | 0.12 | -0.04 |
|  | L | 0.05 | 0.03 | -0.04 |
| Parasubiculum | R | **-0.11^*^** | 0.00 | 0.05 |
|  | L | **-0.12^*^** | -0.11 | -0.08 |
| Presubiculum | R | -0.04 | 0.05 | -0.04 |
|  | L | -0.03 | 0.03 | -0.02 |
| Subiculum | R | -0.01 | 0.03 | 0.01 |
|  | L | -0.01 | 0.00 | 0.01 |
| Hippocampal Fissure | R | 0.00 | 0.02 | 0.10 |
|  | L | 0.04 | 0.03 | 0.02 |
| Hippocampal Tail | R | -0.02 | -0.05 | -0.10 |
|  | L | -0.01 | -0.06 | -0.08 |

SZ, patients with schizophrenia; MDD, patients with major depression disorder; BD, patients with bipolar disorder; CA, cornu ammonis; GC-ML-DG, granule cells in the molecular layer of the dentate gyrus; HATA, hippocampal-amygdaloid-transition-area. T, total; R, right; L, left. Pearson’s *r* is shown.

* *P* < 0.05.

** *P* < 0.01

Table S5

Correlations between drug dose equivalent and hippocampal subfield volumes in SZ, MDD and BD groups.

|  |  | SZ (n=160) | | | MDD (n=69) | | | BD (n=98) | | |
| --- | --- | --- | --- | --- | --- | --- | --- | --- | --- | --- |
|  |  | Antipsychotic | Antidepressant | Lithium | Antiepileptic | Antidepressant | Lithium | Antipsychotic | Antidepressant | Lithium |
| Whole Hippocampus | T | 0.08 | - | - | - | 0.08 | - | 0.08 | - | -0.04 |
|  | R | 0.09 | - | - | - | 0.12 | - | 0.09 | - | -0.02 |
|  | L | 0.07 | - | - | - | 0.03 | - | 0.07 | - | -0.04 |
| CA1 | R | 0.05 | - | - | - | 0.13 | - | 0.06 | - | 0.02 |
|  | L | 0.03 | - | - | - | 0.06 | - | 0.12 | - | -0.11 |
| CA3 | R | 0.05 | - | - | - | 0.16 | - | 0.04 | - | 0.00 |
|  | L | 0.05 | - | - | - | 0.06 | - | 0.06 | - | -0.02 |
| CA4 | R | 0.08 | - | - | - | **0.21^*^** | - | 0.12 | - | -0.03 |
|  | L | 0.05 | - | - | - | 0.09 | - | 0.07 | - | 0.00 |
| Fimbria | R | -0.02 | - | - | - | -0.02 | - | 0.08 | - | 0.10 |
|  | L | -0.03 | - | - | - | 0.07 | - | 0.10 | - | 0.04 |
| GC-ML-DG | R | 0.06 | - | - | - | **0.21^*^** | - | 0.09 | - | -0.03 |
|  | L | 0.05 | - | - | - | 0.06 | - | 0.07 | - | 0.01 |
| HATA | R | -0.02 | - | - | - | 0.05 | - | -0.01 | - | 0.14 |
|  | L | 0.02 | - | - | - | 0.10 | - | -0.05 | - | -0.04 |
| Molecular Layer | R | 0.07 | - | - | - | 0.08 | - | 0.09 | - | -0.07 |
|  | L | 0.05 | - | - | - | 0.03 | - | 0.09 | - | -0.06 |
| Parasubiculum | R | -0.01 | - | - | - | -0.10 | - | 0.05 | - | 0.09 |
|  | L | 0.05 | - | - | - | -0.01 | - | -0.03 | - | 0.00 |
| Presubiculum | R | **0.11^*^** | - | - | - | 0.04 | - | 0.03 | - | 0.03 |
|  | L | 0.09 | - | - | - | 0.00 | - | 0.06 | - | 0.00 |
| Subiculum | R | **0.11^*^** | - | - | - | 0.11 | - | 0.07 | - | -0.02 |
|  | L | **0.12^*^** | - | - | - | 0.01 | - | 0.06 | - | 0.01 |
| Hippocampal Fissure | R | 0.07 | - | - | - | 0.10 | - | 0.00 | - | -0.01 |
|  | L | 0.10 | - | - | - | 0.09 | - | 0.00 | - | -0.03 |
| Hippocampal Tail | R | 0.10 | - | - | - | -0.05 | - | -0.01 | - | -0.03 |
|  | L | 0.05 | - | - | - | -0.01 | - | -0.01 | - | -0.03 |

SZ, patients with schizophrenia; MDD, patients with major depression disorder; BD, patients with bipolar disorder; CA, cornu ammonis; GC-ML-DG, granule cells in the molecular layer of the dentate gyrus; HATA, hippocampal-amygdaloid-transition-area. T, total; R, right; L, left. Pearson’s *r* is shown.

* *P* < 0.05.

** *P* < 0.01.

Table S6

Differences in the laterality of hippocampal subfield volumes between SZ, MDD and BD groups

|  | SZ (n = 204) | MDD (n = 98) | BD (n = 110) | *F_2,405_* | adjusted *P* |
| --- | --- | --- | --- | --- | --- |
| Whole Hippocampus | -0.03 ± 0.04 | -0.03 ± 0.04 | -0.03 ± 0.04 | 0.02 | 0.98 |
| CA1 | -0.04 ± 0.06 | -0.03 ± 0.06 | -0.04 ± 0.06 | 0.56 | 0.57 |
| CA3 | -0.02 ± 0.04 | -0.02 ± 0.04 | -0.02 ± 0.04 | 0.06 | 0.94 |
| CA4 | 0.01 ± 0.09 | 0.03 ± 0.09 | 0.02 ± 0.09 | 1.19 | 0.31 |
| Fimbria | -0.02 ± 0.04 | -0.02 ± 0.04 | -0.02 ± 0.04 | 0.08 | 0.92 |
| GC-ML-DG | 0.01 ± 0.07 | 0.01 ± 0.07 | 0.01 ± 0.07 | 0.08 | 0.92 |
| HATA | -0.01 ± 0.03 | -0.01 ± 0.03 | -0.01 ± 0.03 | 0.74 | 0.48 |
| Molecular Layer | 0.04 ± 0.09 | 0.03 ± 0.09 | 0.04 ± 0.09 | 0.87 | 0.42 |
| Parasubiculum | 0.01 ± 0.03 | 0.01 ± 0.03 | 0.01 ± 0.03 | 1.37 | 0.25 |
| Presubiculum | 0.00 ± 0.04 | 0.00 ± 0.04 | 0.00 ± 0.04 | 1.01 | 0.37 |
| Subiculum | -0.02 ± 0.08 | -0.01 ± 0.08 | -0.02 ± 0.08 | 0.60 | 0.55 |
| Hippocampal Fissure | -0.02 ± 0.04 | -0.02 ± 0.04 | -0.02 ± 0.04 | 0.53 | 0.59 |
| Hippocampal Tail | -0.01 ± 0.02 | -0.01 ± 0.02 | -0.01 ± 0.02 | 0.07 | 0.93 |

Bonferroni correction was applied: adjusted *P* = *P* × 13.

SZ, patients with schizophrenia; MDD, patients with major depression disorder; BD, patients with bipolar disorder; CA, cornu ammonis; GC-ML-DG, granule cells in the molecular layer of the dentate gyrus; HATA, hippocampal-amygdaloid-transition-area. The laterality of hippocampal subfield volumes was calculated using the following formula: Laterality index = (right - left) / (right + left). Means ± SDs are shown. *P* values < 0.05 are shown in boldface.

Table S7

Hemispheric asymmetries (right versus left hemisphere difference) in volume of hippocampal subfield volumes within each of SZ, MDD and BD groups.

|  | SZ (n = 204) | | | MDD (n = 98) | | | BD (n = 110) | | |
| --- | --- | --- | --- | --- | --- | --- | --- | --- | --- |
|  | Mean ± SD | *t* | *P* | Mean ± SD | *t* | *P* | Mean ± SD | *t* | *P* |
| Whole Hippocampus | 87.59 ± 172.65 | 7.25 | **<0.01** | 89.55 ± 163.33 | 5.43 | **<0.01** | 90.25 ± 163.27 | 5.80 | **<0.01** |
| CA1 | 37.97 ± 46.90 | 11.56 | **<0.01** | 34.69 ± 38.70 | 8.87 | **<0.01** | 37.25 ± 52.76 | 7.41 | **<0.01** |
| CA3 | 15.05 ± 28.83 | 7.46 | **<0.01** | 15.64 ± 25.64 | 6.04 | **<0.01** | 18.79 ± 23.37 | 8.44 | **<0.01** |
| CA4 | 9.44 ± 19.69 | 6.85 | **<0.01** | 9.76 ± 18.67 | 5.17 | **<0.01** | 9.77 ± 18.10 | 5.67 | **<0.01** |
| Fimbria | -1.81 ± 16.80 | -1.54 | 0.13 | -5.33 ± 14.19 | -3.72 | **<0.01** | -4.53 ± 17.71 | -2.68 | **<0.01** |
| GC-ML-DG | 11.82 ± 22.75 | 7.42 | **<0.01** | 12.46 ± 22.06 | 5.59 | **<0.01** | 11.96 ± 22.29 | 5.63 | **<0.01** |
| HATA | -0.34 ± 8.70 | -0.56 | 0.58 | -0.93 ± 8.18 | -1.13 | 0.26 | -0.75 ± 7.91 | -0.99 | 0.32 |
| Molecular Layer | 6.10 ± 39.75 | 2.19 | **0.03** | 11.18 ± 39.38 | 2.81 | **<0.01** | 10.90 ± 40.46 | 2.83 | **<0.01** |
| Parasubiculum | -5.20 ± 10.19 | -7.28 | **<0.01** | -3.60 ± 10.60 | -3.36 | **<0.01** | -5.22 ± 10.80 | -5.07 | **<0.01** |
| Presubiculum | -4.76 ± 20.84 | -3.27 | **<0.01** | -6.22 ± 19.23 | -3.20 | **<0.01** | -7.92 ± 19.89 | -4.18 | **<0.01** |
| Subiculum | 0.09 ± 30.21 | 0.04 | 0.97 | 2.04 ± 27.27 | 0.74 | 0.46 | -2.19 ± 29.19 | -0.79 | 0.43 |
| Hippocampal Fissure | 5.54 ± 24.79 | 3.19 | **<0.01** | 2.39 ± 19.58 | 1.21 | 0.23 | 6.01 ± 20.87 | 3.02 | **<0.01** |
| Hippocampal Tail | 19.22 ± 45.97 | 5.97 | **<0.01** | 19.87 ± 44.36 | 4.43 | **<0.01** | 22.18 ± 53.71 | 4.33 | **<0.01** |

SZ, patients with schizophrenia; MDD, patients with major depression disorder; BD, patients with bipolar disorder; CA, cornu ammonis; GC-ML-DG, granule cells in the molecular layer of the dentate gyrus; HATA, hippocampal-amygdaloid-transition-area. Means ± SDs are shown. *P* values < 0.05 are shown in boldface.

Table S8

Correlations between 7 factors of HAMD-24 and hippocampal subfield volumes in MDD group.

|  |  | HAMD-7 | | | | | | | |
| --- | --- | --- | --- | --- | --- | --- | --- | --- | --- |
|  |  | Anxiety/Somatization | Weight | Cognitive_Impairment | Diurnal_Variation | Retardation | Sleep_Disorders | Hopelessness | Total |
| Whole Hippocampus | T | 0.12 | 0.08 | -0.05 | 0.18 | 0.07 | -0.14 | -0.02 | 0.02 |
|  | R | 0.17 | 0.06 | -0.03 | **0.22^*^** | 0.11 | -0.06 | 0.01 | 0.08 |
|  | L | 0.05 | 0.08 | -0.06 | 0.11 | 0.03 | -0.19 | -0.05 | -0.04 |
| CA1 | R | **0.20^*^** | -0.02 | 0.00 | **0.23^*^** | 0.18 | -0.10 | 0.02 | 0.11 |
|  | L | 0.07 | 0.08 | -0.05 | 0.13 | 0.07 | -0.13 | -0.05 | 0.00 |
| CA3 | R | 0.02 | -0.10 | -0.11 | 0.11 | -0.01 | -0.04 | 0.00 | -0.03 |
|  | L | -0.09 | -0.01 | **-0.26^*^** | -0.07 | -0.09 | **-0.31^**^** | -0.15 | **-0.26^*^** |
| CA4 | R | -0.08 | -0.02 | -0.17 | 0.07 | -0.05 | -0.12 | 0.01 | -0.12 |
|  | L | -0.01 | 0.08 | **-0.27^**^** | -0.05 | -0.01 | **-0.30^**^** | -0.12 | -0.20 |
| Fimbria | R | 0.15 | -0.10 | 0.18 | 0.11 | 0.11 | 0.04 | -0.03 | 0.13 |
|  | L | 0.02 | -0.17 | 0.25* | 0.03 | 0.12 | -0.02 | 0.16 | 0.15 |
| GC-ML-DG | R | -0.07 | -0.05 | -0.10 | 0.09 | -0.04 | -0.19 | -0.01 | -0.11 |
|  | L | 0.01 | 0.03 | **-0.24^*^** | -0.04 | -0.02 | **-0.31^**^** | -0.16 | **-0.20^*^** |
| HATA | R | 0.15 | -0.12 | 0.09 | 0.14 | 0.07 | -0.16 | -0.09 | 0.05 |
|  | L | -0.04 | -0.09 | 0.10 | 0.08 | **-0.21^*^** | -0.18 | -0.10 | -0.10 |
| Molecular Layer | R | 0.07 | 0.04 | -0.08 | 0.17 | 0.02 | -0.02 | -0.10 | -0.02 |
|  | L | 0.02 | 0.07 | -0.12 | 0.10 | -0.09 | -0.12 | -0.14 | -0.11 |
| Parasubiculum | R | 0.09 | 0.03 | 0.00 | 0.05 | -0.06 | 0.06 | -0.15 | -0.02 |
|  | L | -0.10 | -0.12 | -0.01 | -0.06 | -0.10 | 0.05 | -0.04 | -0.07 |
| Presubiculum | R | 0.13 | 0.19 | 0.14 | 0.12 | 0.06 | -0.09 | -0.09 | 0.08 |
|  | L | 0.03 | 0.07 | 0.19 | 0.05 | 0.13 | -0.13 | -0.04 | 0.06 |
| Subiculum | R | 0.10 | 0.14 | -0.12 | 0.12 | 0.13 | 0.05 | -0.01 | 0.04 |
|  | L | 0.02 | **0.23^*^** | -0.03 | 0.03 | 0.07 | -0.10 | -0.03 | -0.01 |
| Hippocampal Fissure | R | 0.05 | 0.12 | -0.02 | -0.02 | 0.00 | 0.03 | 0.14 | 0.05 |
|  | L | -0.01 | 0.18 | 0.18 | -0.10 | 0.02 | -0.06 | 0.07 | 0.05 |
| Hippocampal Tail | R | 0.22* | 0.18 | 0.07 | 0.16 | 0.11 | 0.04 | 0.20 | **0.21^*^** |
|  | L | 0.14 | 0.06 | 0.06 | **0.22^*^** | 0.08 | 0.01 | 0.13 | 0.14 |

HAMD, Hamilton Rating Scale for Depression; CA, cornu ammonis; GC-ML-DG, granule cells in the molecular layer of the dentate gyrus; HATA, hippocampal-amygdaloid-transition-area. T, total; R, right; L, left. Pearson’s *r* is shown.

* *P* < 0.05.

** *P* < 0.01.
